# Measurement of Stigmatization towards Adults with Attention Deficit Hyperactivity Disorder

**DOI:** 10.1371/journal.pone.0051755

**Published:** 2012-12-19

**Authors:** Anselm B. M. Fuermaier, Lara Tucha, Janneke Koerts, Anna K. Mueller, Klaus W. Lange, Oliver Tucha

**Affiliations:** 1 Department of Clinical and Developmental Neuropsychology, University of Groningen, Groningen, The Netherlands; 2 Department of Experimental Psychology, University of Regensburg, Regensburg, Germany; Alexander Flemming Biomedical Sciences Research Center, Greece

## Abstract

**Objectives:**

In general, assessment tools for stigma in mental disorders such as attention deficit hyperactivity disorder (ADHD) are lacking. Moreover, misbeliefs and misconceptions about ADHD are common, in particular with regard to the adult form of ADHD. The aim of the present study was to develop a questionnaire measuring stigma in adults with ADHD and to demonstrate its sensitivity.

**Methods:**

A questionnaire initially containing 64 items associated with stigma in adults with ADHD was developed. A total number of 1261 respondents were included in the analyses. The psychometric properties were investigated on a sample of 1033 participants. The sensitivity of the questionnaire was explored on 228 participants consisting of teachers, physicians and control participants.

**Results:**

Thirty-seven items were extracted due to exploratory factor analysis (EFA) and the internal consistency of items. Confirmatory factor analysis (CFA) revealed good psychometric properties of a 6-factor structure. Teachers and physicians differed significantly in their stigmatizing attitudes from control participants.

**Conclusions:**

The present data shed light on various dimensions of stigma in adult ADHD. Reliability and Social Functioning, Malingering and Misuse of Medication, Ability to Take Responsibility, Norm-violating and Externalizing Behavior, Consequences of Diagnostic Disclosure and Etiology represent critical aspects associated with stigmatization.

## Introduction

The core symptoms of attention deficit hyperactivity disorder (ADHD), namely inattention, hyperactivity and impulsivity, result in highly externalized behaviors. This externalized behavior can be easily recognized by the environment and may induce misperceptions and misunderstandings about the condition. Moreover, public perceptions concerning ADHD have been shown to be foremost tied to the impression that ADHD is a disorder mainly seen in white middle-class boys suffering preeminent from symptoms of hyperactivity [1], [2]. This enhances the chance of biasing peoples’ ideas about ADHD to not exist in adulthood. Together with other factors such as a rather unclear etiology of the condition, an increased risk of being stigmatized may result for individuals diagnosed with ADHD. Stigmatization reflects the expression of a discrediting stereotype deriving from falsely assumed associations between a group of people and unfavorable characteristics, attributes or behaviors [3]. Stigmatization can be regarded as to be most aversive for the actual object of stigmatization, which refers to self-stigma and can be described as the individual’s internalization of stigmatizing attributes encountered by the public [4]. However, other forms of stigma than self-stigma, such as public stigma and courtesy-stigma, have been described. Public stigma represents the compliance of a larger community to negative attributes or active denial of characteristics, qualities and rights of the stigmatized target [5]. Courtesy-stigma affects family members or people close to a stigmatized person [6], [7]. Accordingly, family members of a stigmatized person become the focus of stigma due to their mere association with the stigmatized target [6].

Empirical research on stigmatization revealed that not only physical deviances can set individuals apart and trigger stigmatization. Intrinsic characteristics of the individual such as behavioral deviance have also been found to provoke stigma [8]. In this respect, Weiner and colleagues [9] assumed that individuals’ mental or behavioral deviance are even more negatively judged than physical impairments, given their stronger association with uncontrollability and norm-violating behavior in the general public. Referring to ADHD, externalizing and norm-violating behaviors in individuals with ADHD have been shown to be potential sources of stigma that manifest themselves in stereotypes, discrimination, isolation and social rejection [10]–[15]. Moreover, not only behavioral problems linked to ADHD may elicit stigma but also the mere label of ADHD may trigger automatic preconceptions and a tendency for social distance [10], [16], [17]. For example, a study on undergraduate students [15] reported more socially-negative ratings of a young adult person diagnosed with ADHD compared to a person with a medical problem (e.g. asthma) or a person with an ambiguous weakness (e.g. heightened level of perfectionism). Furthermore, in the National Stigma Study-Children [18] adult respondents were less likely to label ADHD as a mental illness and to consider it to be serious in comparison to depression. Martin and colleagues [10] found that adults are less willing to have social contact with children and adolescences with ADHD. Their respondents assumed that ADHD is caused by an ‘incapacity of discipline’ and by a ‘bad character’ which are significant correlates of social distance. In academic settings, children and adolescents with ADHD are more often avoided and negatively appraised by their peers [15] as well as perceived to be both more violent and more likely to behave antisocial [12] than children and adolescents without the condition. Coleman and colleagues [19] revealed that children believe that children with ADHD or depression are to be blamed for their condition because of low effort.

Research demonstrated that stigmatization of individuals with ADHD has adverse consequences leading to diminished self-esteem and self-efficacious beliefs and ultimately a reduced quality of life [8]. Furthermore, it has been shown that stigmatization of pharmacological treatment (e.g. being blamed for loss of control and dependence after adhering to medication) can lead to non-compliance. In this respect, individuals with ADHD try to avoid the labeling by rejecting treatment and ignoring their symptoms [20], [21].

Referring to courtesy-stigma, families, relatives or the social network of the target individual with ADHD can also be affected by stigma. For example, more than three-fourths of parents of children with ADHD reported to have encountered stigmatizing situations [14]. In this context it appears important to consider that stigmatizing experiences (e.g. being blamed for having caused the condition by a specific parenting style) can affect family life immensely [13].

Considering the impact of stigmatization on the various facets of an individual’s life, the sporadic empirical investigation and the shortness of valid assessment tools measuring stigmatization in ADHD is surprising. This is in accordance with findings by Angermeyer and Dietrich [22] who proposed that the significance of stigma in mental illness is in general highly underinvestigated. The majority of studies on stigma associated with ADHD based their results on data derived from questionnaires in response to vignettes depicting fictitious characters suffering from prototypical ADHD symptoms [11], [12], [15] or derived from note taking and observations (qualitative data) during self-help group interventions or clinical interviews [13], [14].

Only a few studies made use of survey responses, most likely because of a lack of psychometrically proven questionnaires for the measurement of stigmatization in ADHD. Kellison and colleagues [23] assessed public stigma associated with ADHD by using the ADHD Stigma Questionnaire (ASQ), a 26-item adaptation of the 40-item HIV Stigma Scale, a questionnaire developed for the assessment of stigma associated with human immunodeficiency virus (HIV) [24]. By omitting the personalized stigma factor, the three-factor structure of the HIV Stigma Scale could be obtained in the ASQ using confirmatory factor analysis (CFA) with the subscales *disclosure concerns*, *negative self-image* and *concerns with public attitudes*. The ASQ represents a valuable contribution in objectifying stigmatizing public beliefs about ADHD and demonstrates psychometric properties of distinct dimensions. However, since it is an adaptation of a disease-specific measure (HIV), no items specifically aiming at stigmatizing beliefs and perceptions related to ADHD are included. For example, the use of medication as a treatment for ADHD is controversially discussed in public and in media [18], [20] but has not been considered in the ASQ. As another example, it is known that persons with ADHD have fewer friends and feel often socially rejected [25], [26].Therefore, perceived social functioning of individuals with ADHD in social interactions are of particular interest. In addition, an assessment tool for the adult population should also cover the level of functioning and reliability in work related settings. Finally, in contrast to HIV, the existence of ADHD is often doubted [10]. Consequently, items examining beliefs about unreliability and aggravation of persons with ADHD appear crucial and should be part of a measure of stigmatization in ADHD.

In conclusion, despite stigmatization in ADHD being an important issue, there is a considerable lack of knowledge. Available measures primarily focus on children or do not distinguish between children, adolescents and adults with ADHD. Since it has only recently been acknowledged that ADHD is a condition that can continue from childhood to adulthood, it can be assumed that the public’s knowledge on ADHD in adults is more limited than their knowledge about childhood ADHD. Therefore studies on and measures for the assessment of stigmatization in adult ADHD are of particular significance. The aim of the present study is to enhance knowledge and conceptual clarity on stigmatization in adults with ADHD. A new questionnaire consisting of statements specifically designed to meet public beliefs and perceptions of ADHD in adulthood is developed. Psychometric properties of the questionnaire are explored and the sensitivity of the questionnaire is assessed by investigating differences between stigma responses of individuals with specific knowledge on ADHD (i.e. teachers, physicians) and individuals without specific knowledge.

## Methods

### Participants

Data were obtained from a total of n = 1261 participants who completed a questionnaire measuring stigma responses towards adults with ADHD. Psychometric properties were explored on a sample of 1033 respondents. 439 of the 1033 respondents were first-year undergraduate psychology students of the University of Groningen, The Netherlands. The remaining participants were recruited via public announcements, word-of-mouth and through contacts of the researchers involved. Respondents’ age ranged from 17 to 79 years with a mean age of 31.3 years (SD = 14.8 years). Mean level of education was 16.4 years (SD = 1.2 years). The sample consisted of 66.0% female and 31.3% male participants with 2.7% missing information. Only 1% of the total sample claimed of never having heard about ADHD and 62.6% of participants stated to know an adult diagnosed with ADHD. On a scale of self-rated knowledge about ADHD ranging from 0 (no knowledge at all) to 10 (expert knowledge), the average score was 4.5 (SD = 2.1). To perform an exploratory factor analysis (EFA) with a subsequent confirmatory factor analysis (CFA), the sample was split into two subsamples (i.e. Subsample 1 and Subsample 2). The allocation of participants to samples has been performed randomly by applying the option of a random selection of cases in SPSS 18. The two subsamples did not differ with regard to their descriptives (Table 1).

**Table 1:** Characteristics of respondents.

	Subsample 1	Subsample 2	Total sample
N	516	517	1033
Sex *(female/male)*	344/158	338/165	682/323
Age *(M±SD in years)*	31.3±14.9	31.3±14.7	31.3±14.8
Education *(M±SD in years)*	16.4±1.2	16.4±1.2	16.4±1.2

A further set of data (n = 228) was collected to demonstrate the sensitivity of the questionnaire by exploring stigma responses of a group of teachers and a group of physicians in comparison to a control group. 77 teachers and 74 physicians were recruited via public announcements, word-of-mouth and through contacts of the researchers involved. All teachers had successfully completed a university study program for teachers and were currently working as teachers for primary or secondary schools. All physicians completed successfully a medicine study program and were currently working as physicians in the health service. A control group (n = 77) was recruited with similar characteristics with regard to age, gender and educational level (all respondents completed high school and received additional training or obtained a university degree) (Table 2).

**Table 2:** Characteristics of teachers, physicians and control participants (n = 228).

	Teachers	Physicians	Controls
N	77	74	77
Sex *(female/male)*	44/33	42/32	44/33
Age *(M±SD in years)*	52.0±9.6	50.6±12.7	52.3±10.9

### Measure

Based on (I) an extensive literature study on social perceptions, myths and stigma in ADHD, (II) the personal clinical experience with adults with ADHD of the researchers involved in this study and (III) patient interviews about their experiences, a deductive approach was applied for initial item generation [27]. Starting point was the generation of a rather large set of items allowing removing of items during the development process [27]. For the item development process, two studies served as theoretical foundation. On the one hand, Kellison and colleagues [23] demonstrated psychometric validity of three subscales of the ADHD Stigma Questionnaire (ASQ), namely *disclosure concerns*, *negative self-image* and *concerns with public attitudes*. On the other hand, Haslam [28] proposed that laypeople conceptualize mental disorders along four dimensions, namely *pathologizing*, *moralizing*, *medicalizing* and *psychologizing*. A total of 64 items has been generated. Items were randomly arranged in the questionnaire to control for possible rang order effects. The questionnaire was constructed using a 6-point-Likert-scale with higher scores indicating higher stigma (−3 = strongly disagree, −2 = disagree, −1 = somewhat disagree, 1 = somewhat agree, 2 = agree, 3 = strongly agree). Prior to the 64 items, eight inventorial questions were asked to obtain general background information including descriptive information of respondents (e.g. age, education). Information about participants’ self-rated knowledge concerning ADHD and their familiarity with adults with ADHD (including personal contact) was obtained at the end of the questionnaire. These questions were introduced at the end to prevent biased responses of participants to the 64 items.

### Procedure

All participants were invited to take part in the study on a voluntary basis. Participants received no reward for participation with the exception of undergraduate students who were credited toward a research requirement. The time to complete the questionnaire was estimated to take around twenty minutes. Participants were informed about the aim of the study and it was emphasized that all data will be analyzed anonymously. The study was approved by the ethics committee of the University of Groningen, The Netherlands.

### Ethics Statement

The study was approved by the Ethical Committee Psychology (ECP) affiliated to the University of Groningen, the Netherlands. Before participating, all participants were informed about the aims of the study. Participants were required to read and acknowledge an information sheet prior to completion of the questionnaire. Formal written consent was not sought for adult participants; submission of completed questionnaires was taken as implied consent. Since participants with an age of 17 years were included (n = 2), written informed consent was obtained from these participants as well as their parents prior to inclusion.

### Data Analysis

#### Exploratory factor analysis

Exploratory factor analysis (EFA) was performed on *Subsample 1* with a subsequent confirmatory factor analysis (CFA) on *Subsample 2* in order to identify distinct factors of stigma in adults with ADHD. For EFA, a principal component analysis (PCA) was applied using orthogonal rotation (Varimax). *Subsample 1* consisted of 516 participants. According to the classification proposed by Comrey and Lee [29], a sample of n ≥500 represents a “*very good”* sample size.

#### Internal consistency

Cronbach’s α was calculated for the total scale and the subscales (factors) of the questionnaire as a measure of internal consistency.

#### Confirmatory factor analysis

To replicate the proposed factor structure model by the EFA on *Subsample 1*, confirmatory factor analysis (CFA) was conducted on *Sample 2* using the program LISREL 8.8 for Windows [30]. *Subsample 2* (n = 517) exceeded the criterion of a minimum sample size of 200 respondents for CFA considerably [27].

The factor structure was examined by the following goodness-of-fit statistics: Chi-Square value with corresponding p-value, normed Chi-Square (χ/df), Root Mean Squared Error of Approximation (RMSEA), 90%-confidence interval of the RMSEA, Standardized Root Mean Square Residual (SRMR) and Comparative Fit Index (CFI).

The *Chi-Square value* with its corresponding p-value belongs to the class of absolute fit indices, measuring how well a specific model fits in comparison to no model at all. The Chi-Square statistics assess the magnitude of discrepancy between the sample and fitted covariance matrices [31]. However, disadvantages of Chi-Square statistics are that both deviations from normality and large sample sizes may result in model rejection [32]. To overcome these disadvantages on Chi-Square interpretations, Wheaton et al. [33] proposed the *normed Chi-Square* which takes the degrees of freedom (χ/df) into account. In the present study, therefore, less weight was given to the Chi-Square test than to the descriptive measure of the normed Chi-Square. The smaller the ratio of the normed Chi-Square the better is the fit of the model. Recommendations for an acceptable ratio range from 5.0 to 2.0 with a *good fit* below a value of 3.0 [27], [32].

The *Root Mean Squared Error of Approximation* (RMSEA) is an indicator of the discrepancy between the model and data covariance matrices per degree of freedom [34]. The consensus about an upper limit of RMSEA is.07 [35]. In addition, a 90%-confidence interval of the RMSEA was calculated. In well-fitting models the upper limit of the confidence interval is less than.08 [32].

The *Standardized Root Mean Square Residual* (SRMR) represents the square root of the difference between the residuals of the sample covariance and the hypothesized covariance model. Values of the SRMR range from 0 to 1 with acceptable models obtaining values up to.08 [31].

The *Comparative Fit Index* (CFI) is a revised version of the Non-Normed Fit Index (NNFI), also known as Tucker-Lewis Index [36]. The CFI compares the sample covariance matrix with a null model which assumes that all latent variables are uncorrelated. The CFI ranges between 0 and 1 with higher values indicating a better fit. There is an agreement that a CFI of ≥.90 to≥.95 indicates a good model fit [31].

The goodness-of-fit statistics of the factor model as proposed in EFA of the present study were compared to the cut-offs and recommendations as cited above. Additionally, fit statistics of the multitrait model were compared to the fit statistics of a single common factor model which served as a competing model. Once the overall fit of the model has been evaluated, each model coefficient was individually examined for its degree of fit. This was achieved by t-tests testing the null hypothesis that the true values of specified factor loadings are zero. All items with non-significant factor loadings (p≤.05) were deleted from further analysis [27].

#### Exploratory analysis of stigma subscales

Stigmatization was analyzed in descriptive statistics on the sample (n = 1033) on the basis of the extracted factor structure. Subsequently, a dependent sample ANOVA with post-hoc pairwise comparisons was performed to assess differences in stigmatization between subscales. The effects of gender and age on stigma were assessed with t-tests for independent samples and Pearson product-moment correlations, respectively. Statistical tests were calculated separately on extracted stigma subscales which led to α-accumulation. To counteract the problem of multiple comparisons, the significance level α was adjusted by using a Bonferroni correction. Moreover, effect sizes (Cohen’s d) were calculated for all comparisons. Following Cohen’s guidelines for interpreting effect sizes [37], negligible effects (d <0.2), small effects (d = 0.2), medium effects (d = 0.5) and large effects (d = 0.8) were distinguished. With respect to correlation analysis, negligible effects (r <0.1) small effects (r = 0.1), medium effects (r = 0.3) and large effects (r = 0.5) were distinguished [37]. Data analysis was performed using SPSS 18 for Windows.

#### Stigma responses of teachers and physicians

Stigmatization of teachers, physicians and control participants was explored on the basis of the extracted factor structure. A multivariate analyses of variance (MANOVA) with post-hoc pairwise comparisons (Scheffé) was calculated to compare stigmatization between the three groups on each subscale and on the total scale. Moreover, effect sizes (Cohen’s d) were calculated for all comparisons. Following Cohen’s guidelines for interpreting effect sizes [37], negligible effects (d <0.20), small effects (d = 0.20), medium effects (d = 0.50) and large effects (d = 0.80) were distinguished.

## Results

### Exploratory Factor Analysis (EFA)

Psychometric properties were assessed by applying EFA on *Subsample 1*. A Kaiser-Meyer-Olkin value of.86 supported sampling adequacy for the analysis and can be categorized as *meritorious* according to Kaiser [38]. The Bartlett’s test of sphericity indicated that correlations between items were sufficiently large for PCA (χ^2^(2016) = 8258.3, p<.001). PCA using orthogonal rotation (Varimax) initially identified 17 components meeting Kaiser’s criterion of an *eigenvalue* >1 which explained 61.1% of the total variance. Items were initially retained when they loaded on a factor with a minimum loading of.40 but did not load to any other factor higher than.39. However, on the basis of a scree plot inspection and calculations of the internal consistency (Cronbach’s α; corrected item-total correlations), it was justified to extract six of the initial components with a total of 37 items for further exploration. All of the six components had *eigenvalue*s ranging from 2.64 to 5.47. Table 3 presents a summary of the PCA, including the rotated factor loadings for each of the 37 items, the obtained *eigenvalues* and the percentage of explained variance for each of the six components. The six components of the PCA were interpreted as the following subscales of the questionnaire: *Reliability and Social Functioning* of adults with ADHD (factor 1), *Malingering and Misuse of Medication* (factor 2), *Ability to Take Responsibility* (factor 3*), Norm-violating and Externalizing Behavior* (factor 4), *Consequences of Diagnostic Disclosure* (factor 5) and *Etiology* of adult ADHD (factor 6).

**Table 3:** Summary of results of the principal component analysis (PCA).

	Factor loadings on each factor					
Item	1	2	3	4	5	6
15. Adults with ADHD care less about other’s problems.	**.66**	.038	.07	.04	.080	<.001
*17. Adults with ADHD are able to take care of a group of children in kindergarten.	**.61**	.07	.30	.15	.16	.06
25. You cannot rely on adults with ADHD.	**.53**	.20	.21	.26	.08	.33
27. Adults with ADHD are self-focused and egoistic.	**.56**	.29	.16	.26	.03	.31
*28. I would go on a date with someone with ADHD.	**.58**	.01	.26	<.01	.11	.06
*32. Adults with ADHD have no problems in making friends.	**.66**	.05	.07	.04	.08	<.01
33. Adults with ADHD are less successful than adults without ADHD.	**.53**	.24	.16	.30	.30	.17
* 35. Adults with ADHD are able to lead a group of people.	**.71**	.05	.20	.07	.08	.03
36. Under medication, adults with ADHD are less trustworthy.	**.65**	.33	<.01	.08	.01	.22
3. Many adults with ADHD simulate the symptoms.	.02	**.67**	.13	.04	.02	.18
4. Adults with ADHD misuse their medication (sell it to others, take too much…)	.16	**.53**	.17	.03	.04	<.01
5. ADHD is invented by drug companies to make profit.	.02	**.42**	.13	.11	.04	.12
7. Many adults with ADHD exaggerate their symptoms in order to be medicated.	.07	**.56**	.11	.03	.09	.19
9. ADHD is a childhood disorder and not seen in adults.	.20	**.49**	.06	.03	.05	<.01
10. Adults with ADHD lie more often than adults without ADHD.	.04	**.60**	.22	.37	.07	.11
11. Adults with ADHD have a lower IQ than adults without ADHD.	.17	**.69**	.10	.24	.08	.11
30. Many adults pretend to have ADHD just to get access to medication.	.28	**.40**	.16	.07	.03	.21
31. Adults with ADHD are less able to give advice.	.32	**.52**	.29	.34	.14	<.01
1. Adults with ADHD are bad parents and have problems with raising children.	.06	.25	**.54**	.18	.09	.29
2. I would mind if my investment advisor had ADHD.	.08	.20	**.71**	.06	.15	.13
*14. I would not mind if a doctor who has ADHD treated me.	.28	.04	**.60**	.05	.02	.18
26. If I had a business, I would not hire a person with an ADHD diagnosis.	.13	.23	**.58**	.22	.04	.05
29. I would mind if the teacher of my children had ADHD.	.21	.17	**.63**	.23	.03	.20
12. Adults with ADHD are more often involved in traffic errors.	.07	.25	.10	**.42**	.20	.05
18. I could tell when a person around me has ADHD.	.14	<.01	.07	**.41**	.06	.04
19. Adults with ADHD act without thinking.	.13	.13	.09	**.66**	.06	.04
20. Adults with ADHD have a different sense of humor than adults without ADHD.	.03	.11	.23	**.55**	.14	.08
37. Adults with ADHD cannot deal with money.	.02	.34	.18	**.44**	.18	.25
6. People’s attitudes about ADHD make persons with ADHD feel worse about themselves.	.21	−.01	<.01	<.01	**.63**	.04
8. Adults with ADHD are of lower social status.	.08	.19	.28	.07	**.58**	<.01
13. As a rule, adults with ADHD feel that telling others that they have ADHD was a mistake.	.11	<.01	.28	.04	**.58**	.08
21. Adults with ADHD have a lower self-esteem than adults without ADHD.	.25	<.01	.01	.31	**.62**	.12
24. Adults with ADHD feel excluded from society.	.15	.08	.05	.12	**.67**	.08
16. ADHD is caused by bad parenthood.	.37	.31	.02	.05	.05	**.43**
22. Extensive exposure to video games and TV shows can cause ADHD.	.17	.08	.06	.08	.07	**.67**
23. Adults with ADHD do not engage enough in sports.	.16	.28	.13	.17	.04	**.60**
34. ADHD is a consequence of childhood trauma.	.13	−.26	.16	.14	<.01	**.41**
***Eigenvalue***	**5.47**	**4.33**	**3.25**	**2.91**	**2.69**	**2.64**
***% explained variance***	**8.55**	**6.77**	**5.01**	**4.55**	**4.20**	**4.12**

N = 516; Cronbach’s α = 0.91; * inversed items; The item numbers reflect the relative position of the items in the original questionnaire.

### Internal Consistency

The overall scale reliability (internal consistency) of the 37 items was high (Cronbach’s α = .91). The scale reliabilities of the six subscales (Cronbach’s α) ranged between.61 and.87, with.60 indicating the minimum acceptable and 0.80 indicating good reliability. Table 4 presents the scale reliabilities of all six extracted components of the PCA.

**Table 4:** Internal Consistency Scales.

Subscales	Number of items	Cronbach’s α (range if items deleted)
1. Reliability and Social Functioning	9	0.87 (0.84–0.86)
2. Malingering and Misuse of Medication	9	0.81 (0.78–0.80)
3. Ability to Take Responsibility	5	0.74 (0.66–0.76)
4. Norm-violating and Externalizing Behavior	5	0.61 (0.52–0.61)
5. Consequences of Diagnostic Disclosure	5	0.65 (0.56–0.62)
6. Etiology	4	0.71 (0.60–0.65)

### Confirmatory Factor Analysis (CFA)

Regarding the overall fit of the model, the Chi-Square statistics led to rejection of the model (χ^2^ (614) = 1763.68; p<.01). More crucial, the normed Chi-Square value was within a range indicating a good fit (χ2/df = 2.87). Both a RMSEA of.06 and its 90%-confidence interval [.057;.064] also pointed to a well-fitting model. The SRMR of.07 was below the recommended cut-off of.08 and therefore further supported the model fit. Finally, the incremental fit index (CFI = .93) also revealed an acceptable model fit. In summary, the CFA resulted in a satisfactory fit for the present 6-factor model. The proposed 6-factor model outperformed a single common factor model (χ^2^ (629) = 2680.78 p<.01; χ^2^/df = 4.3, RMSEA = .099, 90%-CI for RMSEA = [.096;.100]; SRMR = .082; CFI = .87) in all indices. To investigate each model coefficient individually, t-values for factor loadings were inspected. Significant loadings were found for each item (p<.01). Therefore, no items had to be excluded.

### Exploratory Analysis of Stigma Subscales

**Figure 1:**
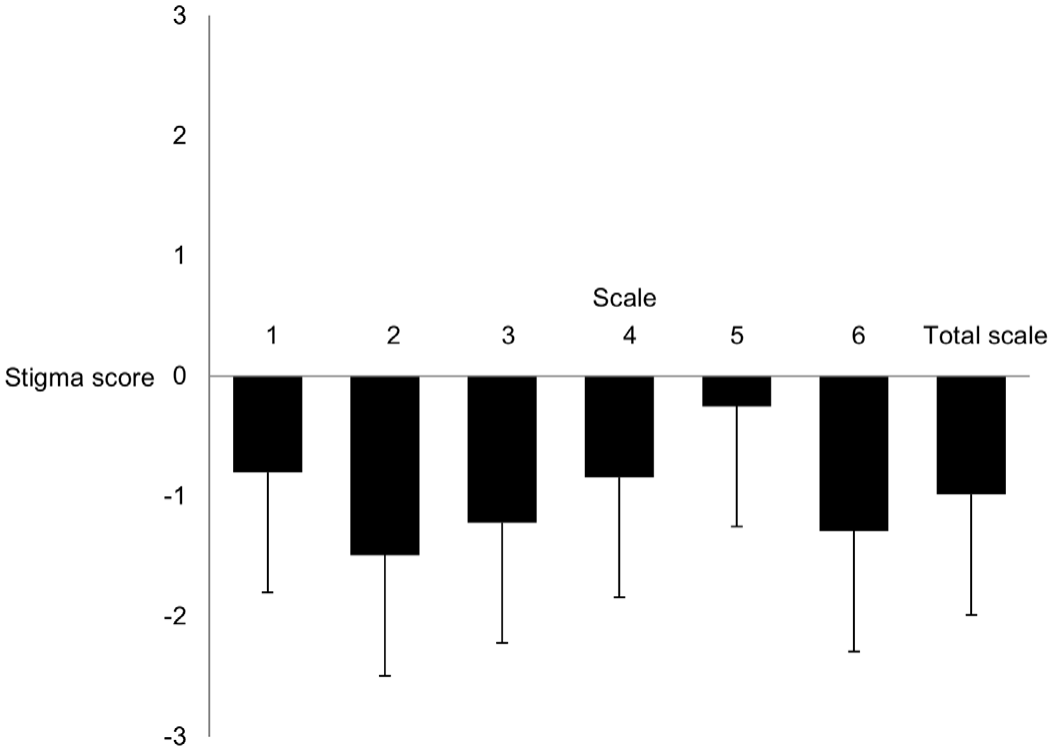
Mean stigma scores and standard deviations on the subscales. Subscale 1: Reliability and Social Functioning; Subscale 2: Malingering and Misuse of Medication; Subscale 3: Ability to Take Responsibility; Subscale 4: Norm-violating and Externalizing Behavior; Subscale 5: Consequences of Diagnostic Disclosure; Subscale 6: Etiology. Higher stigma scores indicate increased stigmatizing beliefs; All subscales differ significantly (p<.05) from each other with the exception of the comparison between 1 and subscale 4 and between subscale 3 and 6 (p>.05).

A dependent sample ANOVA indicated significant overall differences in stigmatization between the subscales (F(5;5095) = 295.2; p<.001). Post-hoc pairwise comparisons revealed significant differences between all pairs of subscales with the exception of the comparison between subscale 1 and subscale 4 and between subscale 3 and subscale 6 (Figure 1). Participants displayed the most pronounced stigma responses on subscale 5 (*Consequences of Diagnostic Disclosure*) followed by subscale 1 (*Reliability and Social Functioning*), subscale 4 (*Norm-violating and Externalizing Behavior*), subscales 3 (*Ability to Take Responsibility*) and 6 (*Etiology*) and finally subscale 2 (*Malingering and Misuse of Medication*). Effect sizes of pairwise comparisons ranged from negligible size to large size (Table 5).

**Table 5:** Effect sizes (Cohen’s d) of differences between stigma subscales.

Subscale	2	3	4	5	6
**1**	0.66	0.36	0.04	0.38	0.44
**2**	*	0.27	0.67	1.10	0.21
**3**		*	0.34	0.77	0.06
**4**			*	0.50	0.37
**5**				*	0.75

Subscale 1: Reliability and Social Functioning; Subscale 2: Malingering and Misuse of Medication; Subscale 3: Ability to Take Responsibility; Subscale 4: Norm-violating and Externalizing Behavior; Subscale 5: Consequences of Diagnostic Disclosure; Subscale 6: Etiology.

For the analysis of the effects of gender and age, a Bonferroni adjusted significance level of p = .0083 was applied because of multiple comparisons/correlations (6 subscales and total score). Stigma scores of female respondents (n = 682; mean age = 30.1 years, SD = 14.3) were compared to the scores of male respondents (n = 323; mean age = 34.5 years, SD = 15.8) using t-tests for independent samples (Table 6). Male respondents had significantly higher stigma scores than female respondents on subscale 2 (t(996) = −3.85, p<.001; d = .26), subscale 3 (t(995) = −4,06 p<.001; d = .27) and subscale 6 (t(994) = −2.76, p = .006; d = .18). The analysis of effect sizes indicated only negligible to small differences. However, male participants were significantly older than female participants (t(1000) = −4.41; p<.001). No significant differences were found on subscale 1 (t(995) = −1.12, p = .26; d = .08), subscale 4 (t(998) = −0.74, p = .46; d = .05) and subscale 5 (t(994) = 0.69, p = .49; d = .05) as well as on the overall stigma score (t(1001) = −2.50, p = .013; d = .16). Furthermore, differences were only of negligible size. Correlational analysis revealed significant associations between age and subscale 1 (r = −0.15, p<.001), subscale 2 (r = −0.20, p<.001) and subscale 3 (r = 0.12, p<.001). According to Cohen [37], these correlations were of small size. The remaining correlations did not reach significance and were only of negligible size (r = 0.01, p = .88 for subscale 4; r = 0.03, p = .38 for subscale 5; r = −0.02, p = .47 for subscale 6 and r = −0.05, p = .12 for the total score).

**Table 6:** Stigma scores (M ± SD) on the subscales and total scale of the questionnaire for male and female respondents (n = 1033).

	Stigma scale						
	1	2	3	4	5	6	Total scale
**Male** (N = 682)	−0.76±1.11	−1.35±0.92*	−1.02±1.16*	−0.81±1.03	−0.28±1.11	−1.15±1.22*	−0.91±0.79
**Female** (N = 323)	−0.85±1.22	−1.57±0.78	−1.31±1.01	−0.86±0.92	−0.23±0.95	−1.36±1.18	−1.03±0.68

Subscale 1: Reliability and Social Functioning; Subscale 2: Malingering and Misuse of Medication; Subscale 3: Ability to Take Responsibility; Subscale 4: Norm-violating and Externalizing Behavior; Subscale 5: Consequences of Diagnostic Disclosure; Subscale 6: Etiology.

### Stigma Responses of Teachers and Physicians

**Figure 2:**
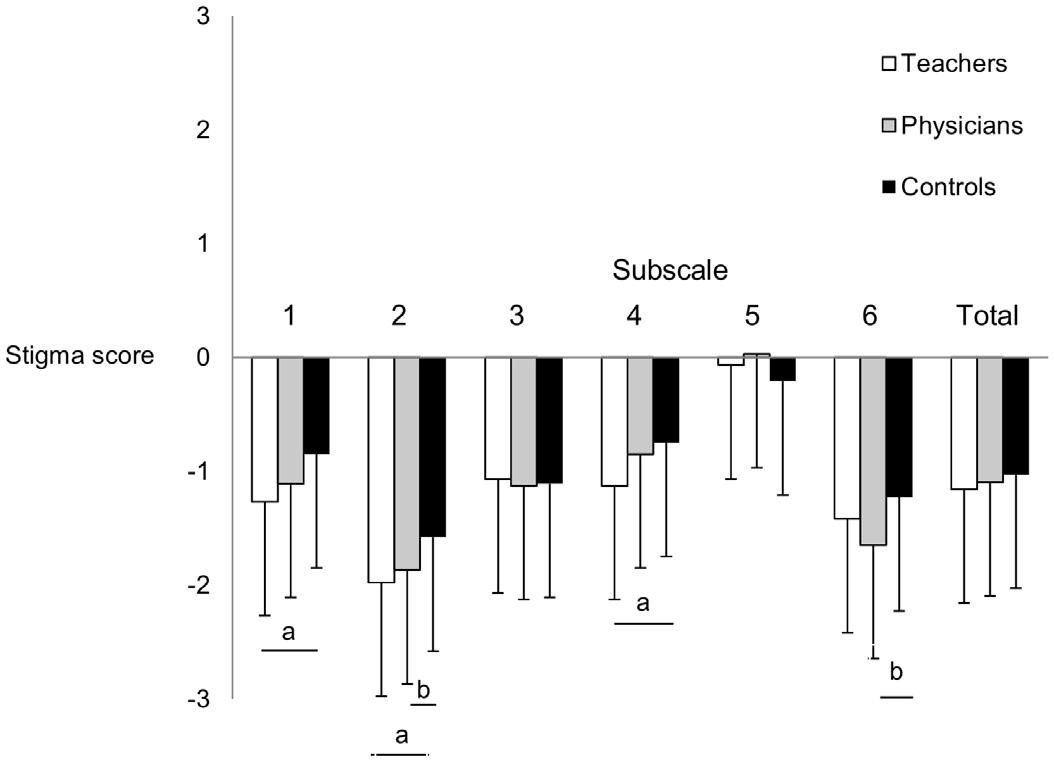
Mean scores of teachers, physicians and controls on each subscale and the total scale. Subscale 1: Reliability and Social Functioning; Subscale 2: Malingering and Misuse of Medication; Subscale 3: Ability to Take Responsibility; Subscale 4: Norm-violating and Externalizing Behavior; Subscale 5: Consequences of Diagnostic Disclosure; Subscale 6: Etiology. ^a^ significant difference between teachers and control participants on p<.05. ^b^ significant difference between physicians and control participants on p<.05.

Stigma responses of teachers, physicians and control participants are presented in table 7. Multivariate analyses of variance (MANOVA) revealed significant group differences on subscale 1 (F(2;225) = 6.10; p = .003), on subscale 2 (F(2;225) = 7.75; p = .001), on subscale 4 (F(2;225) = 3.19; p = .043) and on subscale 6 (F(2;225) = 3.44; p = .034). No significant group differences were obtained on subscale 3 (F(2;225) = 0.06; p = .94), on subscale 5 (F(2;225) = 0.99; p = .38) and on the total scale (F(2;225) = 0.70; p = .50). On subscale 1 and 4, teachers but not physicians expressed significant lower stigma scores than control participants (subscale 1: p = .002; subscale 4: p = .047). On subscale 2, both teachers (p = .001) and physicians (p = .019) indicated significantly lower stigma responses compared to control participants. With regard to subscale 6, physicians but not teachers displayed significantly lower stigma scores than control participants (p = .028) (Figure 2). Effect sizes of stigmatization between teachers, physicians and control participants ranged from negligible to medium size (Table 8).

**Table 7:** Stigma responses of teachers, physicians and control participants.

	Teachers(n = 77)	Physicians(n = 74)	Controls(n = 77)
Subscale 1	−1.27±0.87	−1.11±0.82	−0.85±0.56
Subscale 2	−1.98±0.66	−1.87±0.70	−1.58±0.60
Subscale 3	−1.07±1.27	−1.14±1.24	−1.11±1.10
Subscale 4	−1.13±0.97	−0.85±0.97	−0.75±0.92
Subscale 5	−0.07±0.90	0.03±1.15	−0.21±1.12
Subscale 6	−1.42±0.93	−1.65±1.04	−1.23±0.98
Total Scale	−1.16±0.64	−1.10±0.70	−1.03±0.59

Subscale 1: Reliability and Social Functioning; Subscale 2: Malingering and Misuse of Medication; Subscale 3: Ability to Take Responsibility; Subscale 4: Norm-violating and Externalizing Behavior; Subscale 5: Consequences of Diagnostic Disclosure; Subscale 6: Etiology.

**Table 8:** Effect sizes (Cohen’s d) of stigma responses between groups.

	Teachers vs. Physicians	Teachers vs. Controls	Physicians vs. Controls
Subscale 1	0.19	0.57*	0.37
Subscale 2	0.16	0.63*	0.44*
Subscale 3	0.06	0.03	0.03
Subscale 4	0.29	0.40*	0.11
Subscale 5	0.10	0.14	0.21
Subscale 6	0.23	0.20	0.42*
Total Scale	0.09	0.21	0.11

Subscale 1: Reliability and social functioning; Subscale 2: Malingering and misuse of medication; Subscale 3: Ability to take responsibility; Subscale 4: Norm-violating and externalizing behavior; Subscale 5: Consequences of diagnostic disclosure; Subscale 6: Etiology. * significant at p<.05.

## Discussion

Empirical research directly addressing stigma in ADHD is sparse. One difficulty in studying this topic is the lack of appropriate measures. Therefore, a new questionnaire on stigma in adults with ADHD has been developed in the present study. The aims of this development were (I) to identify dimensions that specifically meet stigmatizing issues persons with ADHD are confronted with, (II) to transfer the knowledge gained from research on childhood ADHD to the adult population, and finally (III) to examine the sensitivity of the questionnaire in measuring differences of stigmatizing attitudes between groups by comparing stigma responses of teachers, physicians and matched control participants.

### Extraction of a Six-factor Structure

The questionnaire consists of 37 items directly addressing stigmatizing beliefs on adults with ADHD. Exploratory factor analysis (EFA) revealed a 6-factor structure which was empirically confirmed by a confirmatory factor analysis (CFA). The devised 6-factor structure has been supported in overall fit indices and by inspecting each model coefficient individually. Furthermore, the 6-factor structure has been shown to be superior to a single common factor model.

Research demonstrated that the mere label of a psychiatric condition can trigger stigmatization [10], [16]. Therefore, items addressing negative self-image of affected individuals and disclosure of the diagnostic status have been included. These items which have been partly adopted from the ASQ [23] loaded on a factor labeled *Consequences of Diagnostic Disclosure*.

Participants’ responses to statements that adults with ADHD are not reliable, less trustworthy, self-focused, egoistic and careless about other’s problems have been found to load on a common factor which has been labeled *Reliability and Social Functioning.* This factor may reflect the difficulties of individuals with ADHD in maintaining social relations, friendships and partnerships [25], [26]. Stigmatizing attitudes as measured by this factor therefore represent a depreciation of the individual’s social abilities and may lead to social rejection and exclusion.

The factor *Ability to Take Responsibility* encompasses statements, which refer to situations in which individuals with ADHD take over the responsibility for another individual, a family or a business (e.g. being a doctor, a parent or an investment adviser). Getting stigmatized on this factor implies doubts about the social, academic and vocational abilities of the person with ADHD. Adverse academic outcomes and adverse career developments might be possible consequences. In fact, it has been shown that persons with ADHD often encounter a range of problems in the occupational setting, comprising peer relational problems accompanied with adverse academic and vocational outcomes [39].

Another important issue in explaining the emergence of stigmatization in ADHD lies in the doubts concerning the existence of ADHD as a disorder [10]. Items in the questionnaire stating that adults with ADHD simulate the symptoms, misuse their medication, pretend to have ADHD to get access to medication or that ADHD is invented by drug companies in order to make profit deal with the objection to acknowledge the existence of ADHD. These items have been empirically shown to represent a common dimension which has been labeled *Malingering and Misuse of Medication.* Stigmatizing believes point to severe doubts about the existence of ADHD in public which can lead to detrimental consequences for affected individuals. For example, the necessity for treatment can be questioned, the access to treatment can be hindered, and help seeking behavior may become discouraged in the individual with ADHD. Furthermore, effective psychosocial and pharmacological intervention strategies may be refused. In conclusion, adverse adherence to and low efficacy of treatment might result.

The factor labeled *Norm-violating and Externalizing Behavior* refers *to* society’s understanding of ADHD which is dominated by the occurrence of behavioral disturbances associated with the condition, in particular with the preeminent hyperactive-impulsive symptoms which are a defining characteristic of childhood ADHD [2]. High agreement on items stating that persons with ADHD act without thinking, cannot deal with money or make more often errors in traffic reflects this notion.

Finally, a dimension has been identified concerning the causes of the condition. This factor has been labeled *Etiology* and implies common stigmatizing beliefs about the etiology of ADHD (e.g. in claiming that adults with ADHD do not engage enough in sports or that extensive exposure to video games and TV shows cause ADHD). The importance of this dimension is indicated by findings showing that knowledge about the etiology of mental disorders, such as ADHD, may adjust stigmatizing beliefs [1], [6].

The present study did not reveal a factor focusing on medication use. This might appear surprising, since misperception and stigmatizing beliefs about long-term effects of medication in ADHD were repeatedly reported [20], [40]. Furthermore, Kellison and colleagues [23] suggested the inclusion of questions about stigma associated with medication into a questionnaire on stigma in ADHD. Therefore, 14 items concerning stigma associated with medication were included in the original version of the questionnaire (64 items), such as items about pharmacological treatment making persons with ADHD less trustworthy and about individuals with ADHD misusing their medication. However, factor analyses did not extract any reliable factor related to stigma associated with medication. In conclusion, the empirically supported structure of the questionnaire provides valuable insight into stigmatization of adults with ADHD. New dimensions of stigmatization were empirically established which are related to various facets including social life, well-being, career development, treatment prospects and an overall life satisfaction.

### Effects of Stigmatization on Stigma Dimensions

The overall level of stigma was found to be low to moderate as reflected in negative values for all subscales. Even though the absolute value is difficult to interpret due to lacking reference values, conclusions can be drawn on the basis of comparisons of stigmatizing responses between subscales. Except of two comparisons (between subscale 1 and subscale 4 and between subscale 3 and subscale 6), stigma responses on all subscales differ significantly from each other. Effect sizes between subscales ranged from negligible to large effects, supporting the notion that different dimensions of stigmatization towards adults with ADHD were measured. The lowest stigma responses could be shown on subscale 2 (*Malingering and Misuse of Medication*). Significant more stigmatization with small effects were found on subscale 3 (*Ability to Take Responsibility*) and subscale 6 (*Etiology*). Representative items for these factors are item 3 for subscale 2 (“*Many adults with ADHD simulate the symptoms”*), item 2 for subscale 3 (“*I would mind if my investment advisor had ADHD”*) and item 23 for subscale 6 (“*Adults with ADHD do not engage enough in sports”*). Stigma responses on these subscales were exceeded by stigma scores on subscale 1 (*Reliability and Social Functioning*), subscale 4 (*Norm-violating and Externalizing Behavior*) and subscale 5 (*Consequences of Diagnostic Disclosure*). These differences were of small to large size. Within these subscales, high stigmatizing responses were observed on item 15 (“*Adults with ADHD care less about other’s problems”*) and item 6 (“*People’s attitudes about ADHD make persons with ADHD feel worse about themselves”*). These results shed light on the trouble persons with ADHD are assumed to have in social life, for example with making friends, being trustworthy, being empathic and caring about other peoples’ problems. These findings refer also to the public’s assumption of socially inappropriate behavior of persons with ADHD which might result in a lower social status. Moreover, the mere diagnostic label and its disclosure is believed to have adverse effects on affected individuals’ self-esteem and general well-being as reflected in high stigmatizing responses. In this context, educational programs aiming to inform the general public about ADHD might be of particular value in reducing the stigmatizing label bias. Previous research already demonstrated that an increase of knowledge about ADHD (e.g. by special training programs) is in general an effective approach in reducing stigmatizing beliefs [41]–[43].

In line with previous research [22], the present study revealed that male respondents expressed more stigmatizing attitudes than female respondents. Men seemed to have more pronounced objections concerning the affected individuals being in responsible functions, as well as more doubts about the concept of both the existence of ADHD and the conventional treatment. However, the value of these findings might be limited since effect sizes were only small and groups differed with regard to age. Indeed, weak relations were observed between age and stigma dimensions in the present study. Older participants showed stronger stigmatizing attitudes on the subscale *Ability to Take Responsibility*. In contrast, younger participants expressed stronger stigmatizing believes on the dimensions *Reliability and Social Functioning* and *Malingering and Misuse of Medication.* The difference in directions of correlations might result from a higher prevalence of medication misuse and simulation within the younger population. For example, external incentives for an ADHD diagnosis are mostly present within the younger population, such as getting access to stimulants or getting improved conditions within educational institutes (e.g. free laptop, access to special bursaries, award of extra time for assignments). This is supported by the finding that an increasing number of students present themselves to specialists at the post-secondary-level because of ADHD symptoms [44]. Furthermore, symptom exaggeration has been found in about 48% of college students who referred themselves to campus-based clinics for ADHD evaluation [45]. Moreover, a shift in focus and priorities from early to late adulthood could be applied as a further explanation. Whereas in early adulthood the emphasis lies on social and academic benefits, the priority may shift to responsibility in later adulthood.

### Stigmatization of Teachers and Physicians Towards Adults with ADHD

When studying stigmatization towards a developmental disorder such as ADHD, people working in the educational or health care sector are of particular interest. Teachers, on the one hand, received a specialized training in educational sciences and have contact to a wide range of youngsters and accompany them in their development from childhood through adolescence into adulthood. Therefore, teachers can be assumed to be more sensitive towards developmental disorders such as ADHD and to be less prone to stigmatizing attitudes towards those affected by these disorders. Physicians, on the other hand, successfully completed a university study program in medicine and have a broad understanding of factors underlying human behavior. Moreover, stigma is a concept that is quite salient in medicine, since individuals with many different physiological, psychosomatic or psychological conditions experience stigmatization. Consequently, physicians have a higher chance to get in contact with people experiencing stigmatization than the general population. Furthermore, many physicians might even be informed by their professional associations, colleagues, conferences or any other additional training about the existence of ADHD in adulthood and the problems involved. Therefore, physicians are presumably also more sensitive with regard to stigmatizing beliefs and attitudes. However, analysis of the present data revealed that teachers, physicians and control participants did not differ on the overall level of stigmatization. Nevertheless, teachers and physicians could be found to show lower scores than control participants on certain aspects of stigma. Significantly lower stigma responses were found for teachers in statements focusing on *Reliability and Social Functioning*, *Malingering and Misuse of Medication* and *Norm-violating and Externalizing Behavior.* Physicians showed significantly less stigmatization on the subscales *Malingering and Misuse of Medication* as well as *Etiology*. Although teachers and physicians did not differ from control participants on the total scale, the present results indicated that teachers and physicians are more sensitive to stigma in ADHD, at least in some aspects. Moreover, different patterns of differences between teachers and physicians in comparison to control participants were found, as indicated by lower stigma of teachers but not physicians on the subscales *Reliability and Social Functioning* and *Norm-violating and Externalizing Behavior.* However, lower stigma responses were found concerning the etiology of ADHD in physicians but not teachers. Considering the educational background of teachers and the expert knowledge of physicians, it is not surprising that physicians were more sensitive with respect to the etiology of ADHD than the general population, while teachers were more sensitive to the functioning of individuals with ADHD. Teachers of course experienced that children with ADHD can learn to cope with their problems and difficulties under certain circumstances, that they have social skills and that they can take over responsibilities. Teaching and mentoring these children over a longer period of time, sometimes even till early adulthood, might provide them with a unique insight in the development of individuals with ADHD. Consequently, teachers might see much more the potential in individuals with ADHD than the general population, making them less prone to stigmatize individuals with ADHD. The different profiles observed therefore most likely reflect the effect of different trainings and experiences between more experienced groups (teachers and physicians) and the general population (control participants). These different profiles, however, also point to the existence of different qualities/dimension of stigmatization. Finally, the detection of these differences also indicates that the questionnaire presented appears to be sensitive with regard to stigmatization of adults with ADHD.

### Limitations and Future Directions

In the present study, a new questionnaire with six subscales assessing stigmatization in adults with ADHD has been described. The psychometric properties of these subscales were carefully evaluated and the factor structure was replicated as suggested by Hinkin [27]. Furthermore, stigma responses from teachers and physicians supported the sensitivity of the questionnaire in measuring stigma towards adults with ADHD. Even though extracted dimensions have been thoroughly named and internal consistency provide support for the factor structure, some items lack in face validity to the loaded dimension. It also has to be pointed out that the six factors only explain a part of the total variance. Therefore, the present instrument should be understood as a “beta version” which needs further development and elaboration. The high number of participants stating to know an adult with ADHD might not be representative for the general population and may therefore represent a limitation of the present findings (i.e. generalization of results). It would be desirable if future research would take these limitations into consideration. In addition, the view of individuals with ADHD themselves would provide valuable information. Comparisons of perceived stigma between individuals with ADHD and the general population appear to be promising. This would allow the specification of public stigma and the evaluation of the individual’s self-perception as a depreciated identity. Furthermore, a comparative investigation of public stigma, self-stigma and courtesy-stigma would be possible. Finally, the ecological validity of surveys in general is often questioned, since the impact of attitudes on the actual behavior is not fully explained. Therefore, it would be desirable to compare the present survey data with data as measured with different assessment tools such as vignettes or videos. This would also provide information about the convergent validity of the questionnaire. In conclusion, the questionnaire presented in this study appears to be a sensitive measure for the assessment of stigmatizing attitudes and beliefs with regard to adults with ADHD. Since there is a scarcity of measures (in particular questionnaires allowing efficient and repeated measurements), this questionnaire might contribute to fill this gap. From the start, this questionnaire was designed to assess stigma in ADHD and therefore represents a disease specific measure. Current data also indicate that *Reliability and Social Functioning*, *Malingering and Misuse of Medication*, *Ability to Take Responsibility, Norm-violating and Externalizing Behavior*, *Consequences of Diagnostic Disclosure* and the *Etiology* of adult ADHD appear to be crucial dimensions when dealing with the construct of stigma in ADHD. The application of the questionnaire may support in the process of the development of effective prevention and intervention strategies against stigmatization of adults with ADHD and persons with related psychiatric conditions.
